# Founder heterozygous P23T CRYGD mutation associated with cerulean (and coralliform) cataract in 2 Saudi families

**Published:** 2009-07-24

**Authors:** Arif O. Khan, Mohammed A. Aldahmesh, Faisal E. Ghadhfan, Saleh Al-Mesfer, Fowzan S. Alkuraya

**Affiliations:** 1 Department of Genetics, King Faisal Specialist Hospital & Research Center, Riyadh, Saudi Arabia; 2 Division of Pediatric Ophthalmology, King Khaled Eye Specialist Hospital, Riyadh, Saudi Arabia; 3 Department of Pediatrics, King Khalid University Hospital and College of Medicine, King Saud University, Riyadh, Saudi Arabia

## Abstract

**Purpose:**

To assess for γD-crystallin (*CRYGD*) mutation in 2 Saudi patients with cerulean cataract and in a brother of one of the patients who had coralliform cataract.

**Methods:**

Patients and all of their available relatives underwent ophthalmic examination and subsequent sequencing of the candidate gene *CRYGD*.

**Results:**

In the first family, a 4-year-old boy with bilateral cerulean cataract and his 6-year-old brother with similar bluish lens discoloration but in a coralliform pattern were heterozygous for the p.P23T CRYGD mutation. Their father and 2 older brothers, all of whom underwent childhood cataract surgery, also harbored the mutation while the 2 asymptomatic immediate family members did not. In the second family, a 7-year-old girl with bilateral cerulean cataract was heterozygous for the same *CRYGD* mutation. Details of her family history were limited. The patients in the two families shared a common disease haplotype.

**Conclusions:**

This first report of p.P23T CRYGD mutation underlying cerulean cataract in the Saudi population strongly supports the mutation’s relation with the phenotype. Coralliform cataract can represent variable expressivity for the same mutation rather than a distinct entity.

## Introduction

Cerulean (“blue-dot”) cataract is an unusual phenotype characterized by dominant early (juvenile) onset of multiple bluish and white opacities predominantly in the lens cortex with occasional radial central lesions [1]. The most commonly-documented cause is heterozygous p.Q155X βB2-crystallin (CRYBB2) mutation, which has been reported in 3 affected families – one from America [2], one from India [3], and one from China [4]. In addition, 2 other heterozygous gene mutations have been associated with cerulean cataract – p.K297R mutation in MAF (v-maf avian musculoaponeurotic fibrosarcoma oncogene homolog) in one Indian family (with variable microcornea) [5] and p.P23T CRYGD mutation in one Moroccan family [6,7]. Heterozygous p.P23T CRYGD mutation has also been associated with coralliform cataract, an even less-frequently reported dominant cataract phenotype which is characterized by generally static central radial lenticular opacities that resemble sea coral [1].  Coralliform cataract due to p.P23T CRYGD mutation has been reported in an affected Caucasian [8] and Chinese family [9]. In addition, 2 other heterozygous *CRYGD* mutations have also been associated with coralliform cataract. In another Chinese family, p.R14C CRYGD mutation segregated with nuclear cataract in some family members and with coralliform cataract in others [10]. In a third Chinese family, heterozygous p.G61C CRYGD mutation segregated with coralliform cataract [11]. To the best of our knowledge mutations in a gene other than *CRYGD* have not been associated with coralliform cataract.

The purpose of this study is to assess for *CRYGD* mutations in 2 Saudi patients with cerulean cataract and in a brother of one of the patients who had coralliform cataract. *CRYGD* was selected as a candidate gene because one patient with cerulean cataract had a brother with coralliform cataract and both types of cataract have been previously-associated with *CRYGD* mutations [6-11].

## Methods

Institutional review board approval was granted for this report. Informed consent was obtained from the 2 participating families, who were not known to be related to each other. Clinical ophthalmic assessment and *CRYGD* sequencing was done on a diagnostic basis for the 2 affected patients, a brother of one of the patients who had coralliform cataract, and all available family members. SNP haplotypes for *CRYGD* were constructed, and *CRYGD* was sequenced in ethnically-matched controls.

### Clinical

Ophthalmic examination consisted of visual acuity, slit-lamp biomicroscopy, intraocular pressure, pupillary examination, ocular motility assessment, fundus examination, and cycloplegic refraction (cyclopentolate 1%).

### Genetic

Genomic DNA was extracted from whole blood anti-coagulated with EDTA using the Purgene Gentra DNA Extraction Kit (Cat. # D-5000; Gentra Systems, Minneapolis, MN) according to the manufacturers instructions. The DNA was quantified spectrophotometrically and stored in aliquots at -20 °C until required.

### PCR amplification and DNA sequencing

PCR amplification was performed on a thermocycler (DNA Engine Tetrad; MJResearch, Inc., Hercules, CA) in a total volume of 25 µl, containing 10 ng DNA, 50 mM KCl, 10 mM Tris-HCl (pH 9.0), 1.5 mM MgCl_2_, 0.1% Triton X-100, 0.25 mM of each dNTP, 0.8 µM of each primer and 0.5 Units of Taq polymerase (D-40724; QIAGEN, Hilden, Germany).  For PCR, an initial denaturation step at 95 ºC for 10 min was followed by 40 cycles of denaturation at 95 ºC for 30 s, with annealing at 59 ºC for 30 s and extension at 72 ºC for 30 s followed by a final extension step of 72 ºC for 10 min.  The primers used for amplification of the coding regions of *CRYGD* are listed in Table 1.  All exons and their intronic boundaries of *CRYGD* were sequenced using an Amersham ET Dye Terminator Cycle Sequencing Kit (Amersham Biosciences, Piscataway, NJ) following the manufacturer’s instructions.  Sequence analysis (3730xl DNA Analyzer; Applied Biosystems, Foster City, CA) was performed using the SeqManII module of the Lasergene (DNA Star Inc., Madison, WI) software package using normal sequence for comparison. A haplotype for *CRYGD* was constructed using intragenic and surrounding SNPs. *CRYGD* sequencing was done in 96 Saudi controls (192 chromosomes).

**Table 1:** *CRYGD* primers.

**Exon ID**	**Forward**	**Reverse**
Exon 1-2	CCCGTGGTCTAGCACAGC	TGCTTATGTGGGGAGCAAAC
Exon 3	CTGTGCTCGGTAATGAGGAG	CCATTTGCCTCGTGTGTG

The primers used for gene sequencing were as shown. The GenBank accession number used for *CRYGD* was U66583.

## RESULTS:

**Figure 1:**
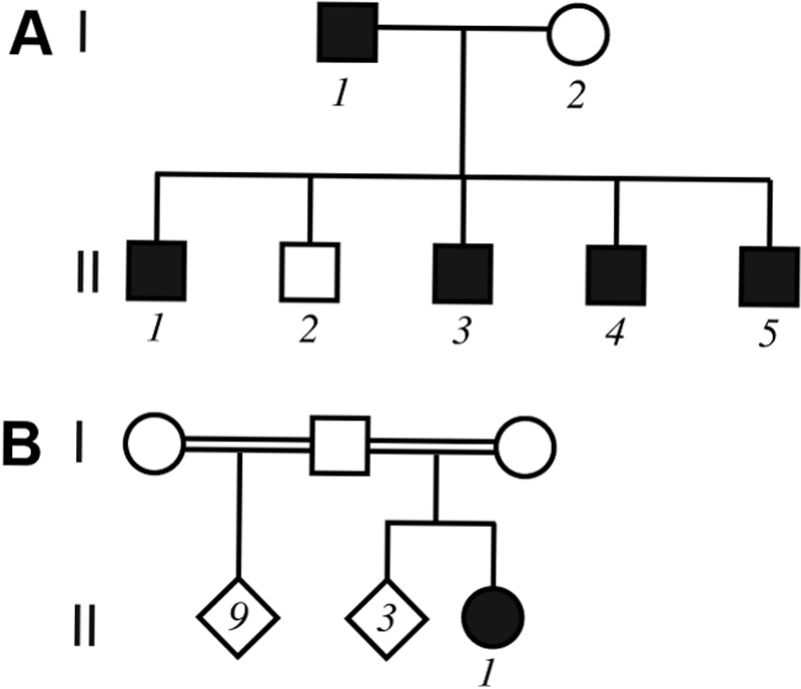
Family pedigrees. The pedigrees of family 1 (**A**) and family 2 (**B**) are shown. Symbols with numbers underneath represent individuals who participated in the study.

**Figure 2:**
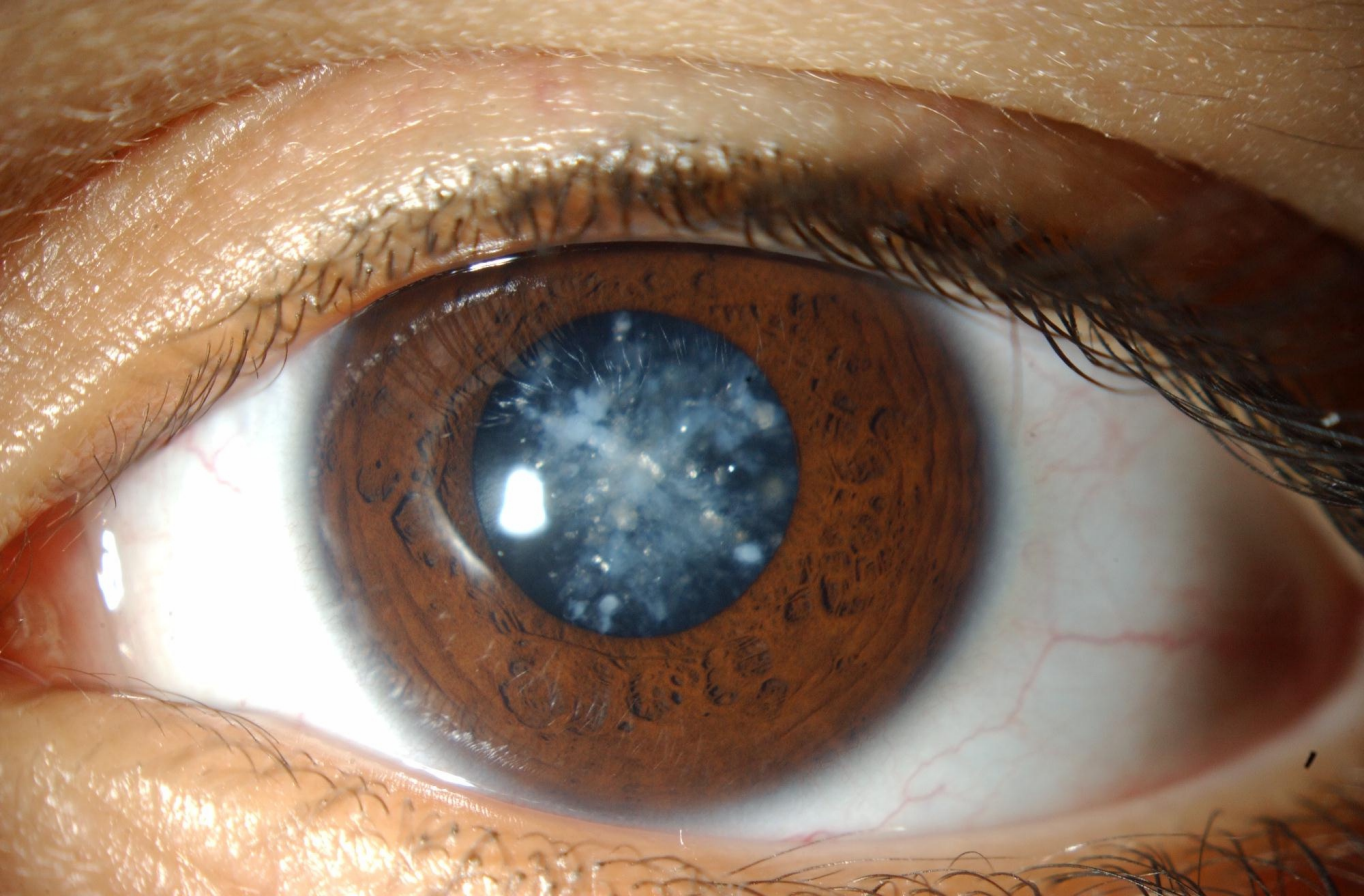
Cerulean cataract in family 1. The left eye of the boy with cerulean cataract is shown. Multiple bluish and white opacities predominantly in the lens cortex with occasional radial central lesions are apparent.

**Figure 3:**
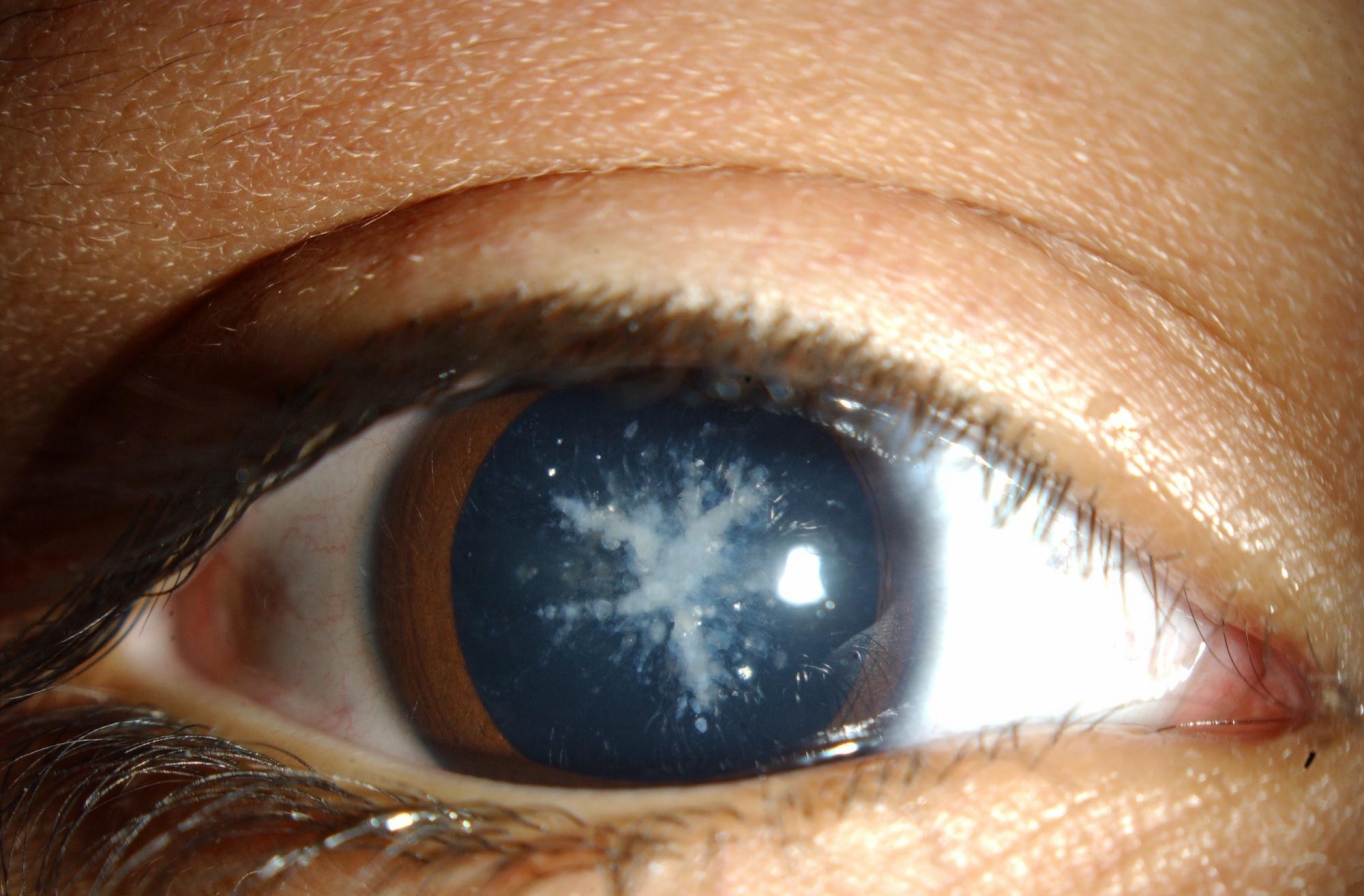
Coralliform cataract in family 1. The right eye of the boy with coralliform cataract is shown (the brother of the patient from Figure 2). Central radial lenticular opacities with a resemblance to sea coral are apparent.

**Figure 4:**
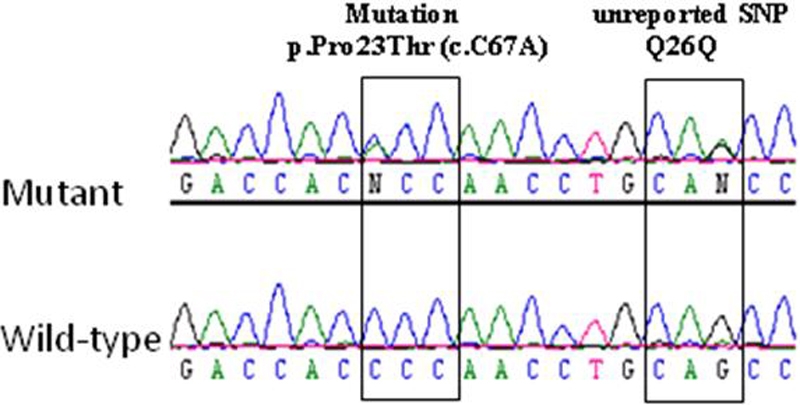
DNA chromatograms of family 1. *CRYGD* sequence chromatograms from the first family highlight the mutation and a novel silent SNP.

Family 1 (Figure 1A): A 4-year-old boy with no other history of medical disease was examined because of worsening photophobia. There was a family history of cataract surgery for juvenile cataract in 2 older brothers and the father. A 6-year-old brother similarly complained of worsening photophobia while the mother and other older brother were asymptomatic. The parents were not consanguineous. Ophthalmic examination of the referred child (II.5) was significant for bilateral cerulean (“blue-dot”) cataract (Figure 2) with approximately 20/40 visual acuity in either eye. His symptomatic brother (II.4) had a medical history significant for difficult delivery, cerebral palsy, difficulty hearing, and developmental delay. Vision could not be quantified because of developmental delay.  Ophthalmic examination was significant for bilateral bluish lens discoloration like in his brother but in a coralliform pattern (Figure 3). Ophthalmic examination of the individuals with a history of childhood cataract surgery (I.1, II.1, and II.3) was significant for bilateral pseudophakia. Ophthalmic examination of the asymptomatic individuals (I.2 and II.2) was unremarkable. *CRYGD* sequencing revealed heterozygous p.P23T CRYGD mutation (c.C67A) in the 2 boys with cataract (II.4 and II.5) and the 3 pseudophakic individuals (I.1, II.1, and II.3) [Figure 4]. The mutation was not detected in the 2 asymptomatic individuals (I.2, II.2).

**Figure 5:**
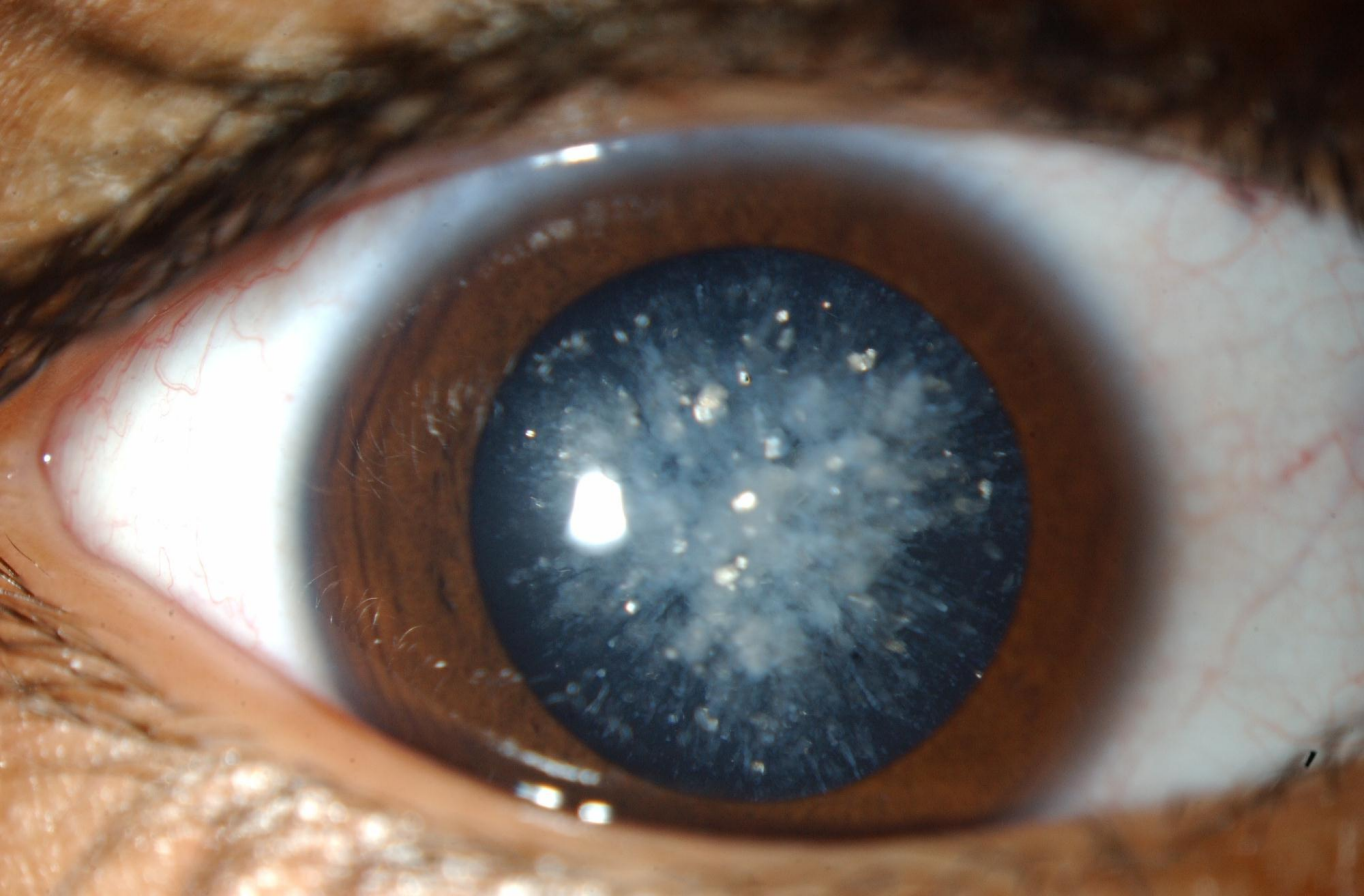
Cerulean cataract in family 2. The right eye of the girl with cerulean cataract is shown. Multiple bluish and white opacities predominantly in the lens cortex with occasional radial central lesions are apparent.

Family 2 (Figure 1B): A 7-year-old girl had been diagnosed with visually-significant congenital cataract soon after birth but never underwent treatment. There was no other history of medical disease. The mother was unaware of any family history of childhood eye disease. According to the mother all siblings of the referred child (3 siblings and 9 step-siblings) were asymptomatic. The parents were first cousins. Ophthalmic examination of the referred child (II.5) was significant for bilateral cerulean cataract (Figure 5) with visual acuity of 20/400 in either eye. Only the referred child was available for ophthalmic evaluation and *CRYGD* sequencing, which revealed heterozygous p.P23T mutation (c.C67A).

Analysis of SNPs flanking *CRYGD* revealed a common disease haplotype for the 2 families (Table 2). *CRYGD* sequencing of controls from the Saudi population (192 chromosomes) was negative for the p.P23T variant.

**Table 2:** Haplotype analysis.

**Family**	**Individual's Status**	**rs11884096 C>T**	**rs966931 C>T**	**rs966932 A>G**	**rs6435415 A>G**	**rs2305429 A>G**	**rs2305430 A>G**	**New SNP Q27Q G>A**
First Family	Affected Father	C/C	C/C	A/A	G/G	A/G	A/G	G/A
	Unaffected Mother	C/C	C/C	G/G	A/A	G/G	G/G	G/G
	Affected Son	C/C	C/C	A/G	G/A	A/G	A/G	G/A
	Unaffected Son	C/C	C/C	A/G	G/A	G/G	G/G	G/G
	Affected Son	C/C	C/C	A/G	G/A	A/G	A/G	G/A
	Affected Son	C/C	C/C	A/G	G/A	A/G	A/G	G/A
	Affected Son	C/C	C/C	A/G	G/A	A/G	A/G	G/A
Second Family	Affected Daughter	C/C	C/C	A/A	G/G	A/G	A/G	G/G
Disease Haplotype		C	C	A	G	A	A	G

Analysis of SNPs surrounding *CRYGD* in the 2 families revealed a common disease haplotype for the 2 families. The disease haplotype (bottom row) can be inferred from the findings in the unaffected mother of family 1.

## Discussion

Heterozygous p.P23T CRYGD mutation has been reported as a cause of cerulean cataract only once previously, in a single Moroccan family [6,7]. The identification of this mutation in 2 Saudi families with cerulean cataract and a common disease haplotype is further evidence for the p.P23T variant as a cause for this rare phenotype. In addition, the finding of heterozygous p.P23T mutation in the patient’s brother with coralliform cataract suggests coralliform cataract and cerulean cataract can represent forms of variable expressivity rather than distinct clinical entities. This idea is also supported by 2 previous reports that similarly described the p.P23T CRYGD mutation as a cause for coralliform cataract [8,9]. Although the phenotypic description of the Moroccan family with cerulean cataract specifically denied that any individual in the family had coralliform cataract, careful inspection of Figure 1B in that report reveals what appears to be a coralliform cataract [7].

Heterozygous p.P23T CRYGD mutation apparently has several measurable effects on γD-crystallin but without significant loss in stability of the protein [6,8,12,13]. Solubility experiments show that the p.P23T mutant has significantly decreased solubility when compared to wild-type γD-crystallin [12,13]. The physical basis for this may relate to the position of Pro23 on the edge of one of the β-sheets of γD-crystallin. Proline in the edge strand of a β -sandwich minimizes the potential for edge-to-edge aggregation events; therefore, its loss in the p.P23T mutant would be expected to lead to increased precipitation of the protein [12,13]. Other changes in secondary structure of the p.P23T mutant include a small decrease in residues in turn conformations with a commensurate increase in β-sheet contents [12]. Despite the decrease in solubility and local alteration of secondary structure, the p.P23T mutant does not have an appreciable loss of stability [12,13]. The absence of the p.P23T variant in our controls and evolutionary analysis of position 23 in the predicted protein [14] both support the pathogenecity of p.P23T CRYGD mutation.

Both intrafamilial and interfamilial phenotypic variability in autosomal dominant cataract for the same genetic mutation is a recognized phenomenon [1]. In addition to cerulean cataract and coralliform cataract, the heterozygous p.P23T CRYGD mutation has been associated with lamellar cataract in an Indian family [15] and fasciculiform cataract in a Chinese family [16]. The most common cause of cerulean cataract – heterozygous p.Q155X CRYBB2 mutation – has also been associated with Coppock-like cataract in a Swiss family [17], progressive polymorphic cataract in a Chinese family [18], and cataract with marked intrafamilial variability in a Chilean family [19]. The other reported cause of cerulean cataract (with variable microcornea) – a heterozygous MAF mutation (p.K297R) – has not been associated with another phenotype to the best of our knowledge. Regarding coralliform cataract, p.R14C CRYGD mutation has also been associated with progressive punctuate juvenile cataract (in a family of unspecified ethnicity) [20] while the p.G61C variant has not been associated with another phenotype to the best of our knowledge. The interaction of background environmental and/or genetic factors with a given crystalline mutation is what leads to a final lens phenotype.

This report of p.P23T CRYGD mutation underlying cerulean cataract in the Saudi population is the second time the association has been described [6,7] and thus supports causation of the phenotype by the genotype. The finding of the variant in the patient’s brother with coralliform cataract is the third time the p.P23T CRYGD mutation has been associated with coralliform cataract [8,9] and supports the concept of coralliform and cerulean cataract  as forms of variable expressivity rather than distinct entities.
